# Correlates of Climate Variability and Dengue Fever in Two Metropolitan Cities in Bangladesh

**DOI:** 10.7759/cureus.3398

**Published:** 2018-10-01

**Authors:** Mohammad Zahirul Islam, Shannon Rutherford, Dung Phung, Md. Nazim Uzzaman, Scott Baum, M. Mamun Huda, Muhammad Asaduzzaman, Mohammad Radwanur Rahman Talukder, Cordia Chu

**Affiliations:** 1 Preventive Medicine, International Center for Diarrhoeal Disease Research, Dhaka, BGD; 2 Epidemiology and Public Health, Griffith University, Brisbane, AUS; 3 Epidemiology and Public Health, International Center for Diarrhoeal Disease Research, Dhaka, BGD; 4 Miscellaneous, Griffith University, Brisbane, AUS; 5 Epidemiology and Public Health, University of Queensland, Brisbane, AUS; 6 Epidemiology and Public Health, Baker Heart and Diabetes Institute, Alice Springs, AUS

**Keywords:** dengue, climate variability, bangladesh, time series analysis, tropical disease

## Abstract

Dengue fever is a major public health concern in Bangladesh with increased incidence during monsoon. We aimed to assess the correlation of temperature, humidity, and rainfall on dengue fever in two dengue endemic cities in Bangladesh. It was a time series analysis of climate factors and dengue occurrence data in Dhaka and Chittagong cities from 1 January 2000 to 31 December 2009. Daily mean temperature, rainfall, and humidity data were obtained from the Bangladesh meteorological department and daily dengue cases data were obtained from the directorate general of health services (DGHS) of Bangladesh. The mean dengue incidence was 31.62 (SD 28.7) per 100,000 in Dhaka whereas it was 5.76 (SD 11.7) per 100,000 population in Chittagong. The incidence of dengue cases was found significantly associated with the monthly mean temperature, total rainfall, and mean humidity in Dhaka, though in Chittagong, the significantly associated factors​ ​​​​​​were monthly total rainfall and mean humidity. The autoregressive integrated moving average (ARIMA) model identified monthly mean humidity and total rainfall as the most significant contributing factors for dengue cases in Dhaka and Chittagong, respectively. Our study reinforces the relationship of climate parameters with dengue fever, which will support policy-makers in developing a climate-based early warning system for dengue in Bangladesh.

## Introduction

The global occurrence of dengue has increased radically in recent decades, endangering about half of the world's population at risk [1]. Approximately 96 million people are clinically manifested by dengue infections per year worldwide and mostly found in urban and semi-urban areas in tropical and sub-tropical climates [2]. Dengue is a climate-responsive disease and climate factors like temperature and rainfall are the most important factors in the occurrence and transmission of dengue fever [3]. Bangladesh is in the South-East Asia region of WHO, which is characterized by strong seasonal variation and heavy monsoon rainfall [4]. Dengue is highly seasonal in Bangladesh with an increased incidence during the monsoon [5]. Although the first recognized outbreak of dengue in Dhaka, the capital city of Bangladesh, was recorded in 1964 [6], followed by sporadic cases of dengue fever during 1977–78 and 1996–1997 [7-8], the extent of dengue occurrence in Bangladesh was poorly documented. The first identified epidemic of dengue fever in Bangladesh took place during the monsoon season of 2000 and resulted in 5,521 officially reported cases, with 93 fatalities [9-10]. From 2000–2009, 91% of all reported dengue cases were from Dhaka, making it the most endemic urban area of the country [11]. However, dengue is also spreading to other urban cities in Bangladesh. Several studies have been conducted to see the climatic variability and spread of dengue cases in Dhaka city [12-14], but very little attention has been given in other cities in Bangladesh. Therefore, we aimed to conduct a study on climatic variability and its association with the distribution of dengue cases in not only Dhaka city but also in other urban cities in Bangladesh. Chittagong is the second largest urban city on the southeastern coast of Bangladesh, which is also endemic for dengue [15]. To our knowledge, this is the first study that aims to assess the correlation of temperature, humidity, and rainfall on dengue fever in two major urban cities (Dhaka and Chittagong) in Bangladesh.

## Materials and methods

The study was conducted in Dhaka and Chittagong cities of Bangladesh. Dhaka is the capital and the largest city of Bangladesh. It has a hot and humid tropical climate with an annual average mean temperature of approximately 26.1°C [16], which is optimal for the development, longevity, and fecundity of *Aedes aegypti* mosquito [17]. Rainfall is highly seasonal and occurs from May to September, and nearly 80% of the annual average total rainfall of 170 mm falls during the monsoon. The annual average mean relative humidity of Dhaka is 65.1% [18]. Dhaka has been the site of dengue outbreaks, and it represents an ideal place for considering the impact of climate variability in relation to dengue fever [5]. Chittagong city is bounded to the east by hills merging with the sea to the west. The annual average mean temperature and total rainfall are 25.9°C and 249 mm, respectively [19]. The warm and wet season starts from April and continues up to October during which mosquito-borne diseases increase significantly. The annual average mean relative humidity is recorded as 73.2%, which helps the parasite to complete the necessary life cycle to transmit the infection [20]. Outbreaks of dengue fever in the last few years in this urban area [15] is the key reason to choose this area as a study site.

It was a time series analysis of climate parameters and dengue occurrence data from January 1, 2000, to December 31, 2009, in two urban cities in Bangladesh.

The daily average maximum and minimum temperatures, average rainfall, and relative humidity data were obtained from the Bangladesh meteorological department over a period of 10 years (2000-2009) for both Dhaka and Chittagong cities. Daily dengue fever data were obtained from the directorate general of health services (DGHS) of the ministry of health and family welfare (MOH&FW) for the same duration. The total number of dengue fever cases was 22,970 during the study period of which 21,748 and 1,222 were in Dhaka and Chittagong, respectively.

All hospitals of those two cities, which were mostly government hospitals, sent their monthly dengue case report as suspected or probable dengue fever (DF) to the DGHS office. Suspected cases were those who had clinical features only and probable cases were those who had positive serological test along with clinical features. No confirmatory procedure was followed in the case detection stage although virus isolation was the confirmatory test. This study included both primary and secondary dengue infection. The physicians in each hospital diagnosed dengue fever from those patients who were admitted with acute febrile illness following a clinical case definition of dengue fever, as mentioned in the WHO guideline for dengue fever [21].

The study was approved by the research ethics committee of Griffith University, Australia. The dengue fever cases were de-identified to maintain privacy.

Means and standard deviations of monthly climatic data were calculated and compared for both study sites. The differences in mean temperature, mean humidity, and total rainfall between the cities were examined using a nonparametric Mann Whitney U-test. A time series plot was used to show the trends in the annual average mean temperature, mean humidity, and total rainfall for the period 2000 to 2009. The Spearman correlation coefficient was used to examine the relationship between dengue cases and each of the climatic variables - rainfall, temperature, and humidity per month. The autoregressive integrated moving average (ARIMA) regression model was used to examine the independent relationship between dengue cases and each climatic variable per month. The dependent variable for the ARIMA model was dengue cases per month whereas the independent variables were mean monthly temperature, mean monthly humidity, and total monthly rainfall. The expert modeler available in SPSS statistical software (version 17) (SPSS Inc., Chicago, IL, USA) was used to determine the best-fitting ARIMA model for Dhaka and Chittagong cities. The stationary R-squared and Ljung Box Q tests were examined and reported to show the goodness of fit of the best-fitting ARIMA model. A p-value less than 0.05 was considered statistically significant. Data were analyzed using SPSS software version 17.

## Results

Trends of climate variables

**Figure 1:**
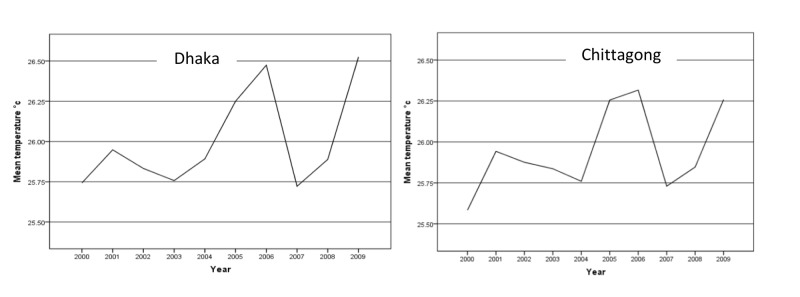
Annual average mean temperature in Dhaka and Chittagong city from 2000 to 2009

A similar increasing trend of annual average mean temperature was observed for both the cities, with a peak in 2006. The mean temperature rose by 0.7˚C in Dhaka and 0.6˚C in Chittagong over the period 2000 to 2009. The lowest mean temperature in Dhaka was recorded as 25.7˚C in the years 2000, 2003, and 2007 and 25.6˚C in Chittagong in the year 2000 (Figure 1).

**Figure 2:**
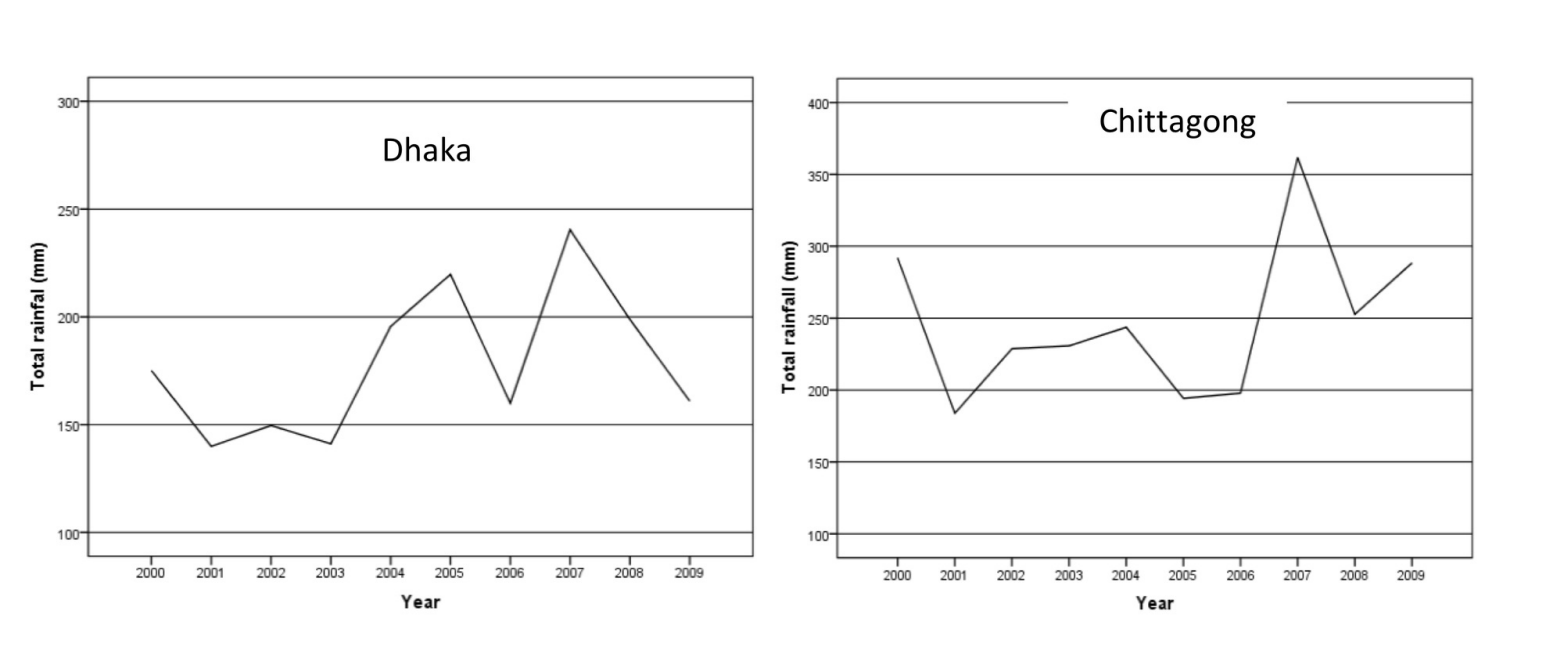
Annual average total rainfall in Dhaka and Chittagong city from 2000 to 2009

The annual average total rainfall was higher in Chittagong as compared to Dhaka. The average total rainfall in Dhaka was 150-200 mm till 2004 but became more variable for the rest of the study period. The highest rainfall of 250 mm in Dhaka was recorded in the year 2007. On the other hand, the average total rainfall in Chittagong was 200-300 mm from 2002 to 2006 but observed many variations during other years. The highest rainfall of 350 mm in Chittagong was recorded in the year 2007 (Figure 2).

**Figure 3:**
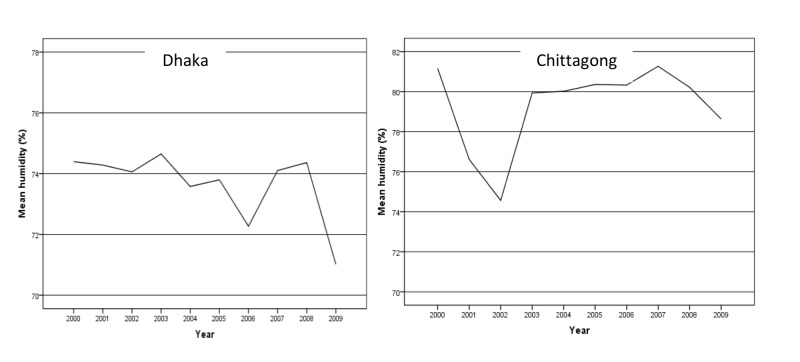
Annual average mean humidity in Dhaka and Chittagong city from 2000 to 2009

The annual average mean humidity in Chittagong was higher than in Dhaka. The mean humidity was between 72% and 76% in Dhaka except in 2009 while in Chittagong, it was around 80% except in 2002 (Figure 3).

There was no statistically significant difference in terms of the mean temperature between Dhaka and Chittagong. However, the total rainfall and mean humidity were found statistically significant between the cities whereas the Spearman correlation analysis showed that in Dhaka, dengue cases were statistically significantly (P-value <0.05) associated with all the key climate variables (temperature, rainfall, and humidity) whereas in Chittagong, rainfall and humidity were statistically significantly associated (Table 1).

**Table 1:** Comparison and correlation between dengue cases and climate variables of Dhaka and Chittagong from 2000 to 2009 r = correlation coefficient

Climate variables	Dhaka	Chittagong	P-value	Dhaka	Chittagong
	Mean (SD)	Mean (SD)		r (P)	r (P)
Temperature in °C	26.0 (0.30)	25.9 (0.25)	0.762	0.300 (0.001)	0.137 (<0.135)
Rainfall in mm	178.1 (34.4)	247.38 (54.5)	0.005	0.356 (<0.0001)	0.307 (0.001)
Humidity in %	73.65 (1.14)	79.31 (2.14)	<0.0001	0.574 (<0.0001)	0.409 (<0.0001)

Trends of dengue fever

**Figure 4:**
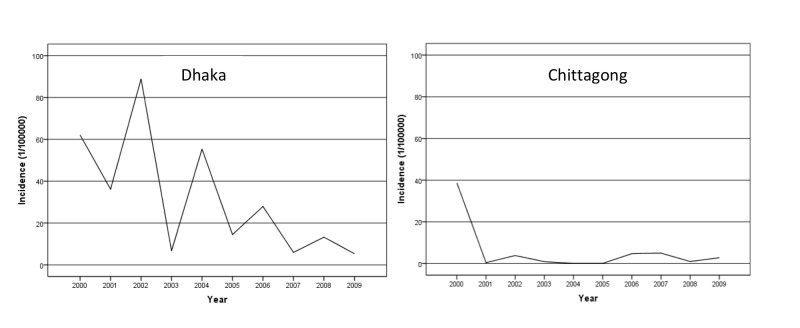
Annual incidence of dengue cases in Dhaka and Chittagong city from 2000 to 2009

The annual incidence of dengue cases varied substantially between the two cites. The mean dengue incidence was 31.62 (SD 28.7) per 100,000 in Dhaka whereas the mean dengue incidence in Chittagong was 5.76 (SD 11.7) per 100,000 population. The incidence in Chittagong was lower than in Dhaka. The numbers and rates of dengue cases varied significantly from year to year in Dhaka and have decreased gradually, whereas in Chittagong rates have been consistently low since 2001 (Figure 4).

**Figure 5:**
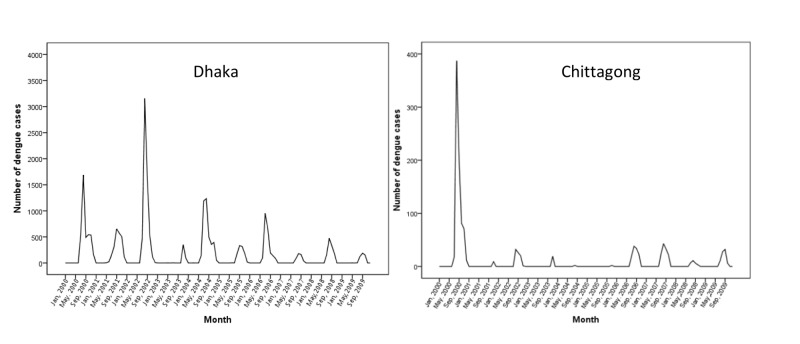
Monthly distribution of dengue cases in Dhaka and Chittagong city from 2000 to 2009

In 2002, the highest number of dengue cases reached more than 3,000 in Dhaka whereas in Chittagong, it was around 400 in the year 2000 (Figure 5).

The autoregressive integrated moving average (ARIMA) model demonstrated that the monthly average dengue cases were significantly associated with the mean monthly humidity in Dhaka up to three-months lags. The association was positive at lag 0 (P<0.001) and 1 (P<0.001) month lag, but it was negative at two months lag (P<0.001) and for Chittagong, the model showed that the monthly total rainfall was significantly (P-value<0.05) associated with monthly dengue cases at the 0 month lag only (Table 2).

**Table 2:** The autoregressive integrated moving average (ARIMA) model parameters

City	Climate variable	Lag (month)	Estimate (SE)	P-value	Stationary R-squared	Ljung Box Q (18)
Dhaka	Mean humidity	Lag 0 (month)	5.697 (0.877)	<0.001		
		Lag 1 (month)	1.276 (0.053)	<0.001	0.935	0.74
		Lag 2 (month)	-0.866 (0.051)	<0.001		
Chittagong	Total rainfall	Lag 0 (month)	0.004 (0.002)	0.023	0.984	0.562

## Discussion

This study provides some evidence of the climate-sensitivity of dengue fever outbreaks and the vulnerability to the health consequences of climate change in Bangladesh. It has been identified that dengue rates co-vary with climatic patterns [22], however, the relation between climatic patterns and dengue fever is not well understood [23]. This is because of the complexity of the life cycle of the vector and the host. The life cycle, maturation, and breeding pattern of the *Aedes aegypti* vector is clearly climate sensitive, and is also influenced by a range of meteorological or related factors, not just the three common features of "weather," but also relative humidity, solar radiation, wind direction and speed, ocean temperatures, and some other factors, eg, environment, waste, etc. [24]. However, from the point of view of the current study, the main shortfall is the lack of insight into the status of the climate-dengue link in the context of Bangladesh. A thorough literature review was carried out on climate change and the transmission of vector-borne diseases, including dengue variants with their inclusion criteria. Very few studies were found in this regard [25], although Bangladesh has all the favorable conditions for dengue to thrive. Since that review, there have been at least two studies identified that partly addressed this gap. The seasonal autoregressive integrated moving average (SARIMA) models for Dhaka did not take account of climate variables per se, but instead seasonality, which, naturally, inherently captures key climatic variables [26]. In another study in Bangladesh, a model that includes a range of climatic factors (monthly humidity, rainfall, and minimum and maximum temperature) was built and found that climatic factors, in particular, rainfall, temperature, and relative humidity did significantly predict monthly dengue occurrence [7]. Furthermore, the seasonality of dengue cases and the seasonality of rainfall and temperature were found largely consistent across the years in Dhaka city [13]. Our study found that the mean monthly rainfall and humidity were statistically significant for both the cities but the monthly mean temperature was significant only for Dhaka.

Moreover, this study attempts to look into the climate parameters of dengue in the large cities for a decade. But, the lack of data or the poor record-keeping system of different strata of population vulnerability (eg. gender differences, age difference, socio-economic difference, rural versus urban, etc.) are a key gap. The irregular or poor quality data affected the extent and comprehensiveness of the analysis of dengue data for Chittagong and Dhaka too. There were more missing data or incomplete data in Chittagong than in Dhaka, which indicates a poor data record-keeping and reporting system in Chittagong. It may be due to a weak monitoring system or a lack of awareness among the reporting authorities.

## Conclusions

This study reinforces the relationship of climate variability with dengue fever in two big cities, Dhaka and Chittagong, in Bangladesh. The annual average rainfall and humidity were significantly associated with dengue occurrence in both cities whereas annual average temperature was found significant factor only in Dhaka. The study findings will be helpful for policy-makers and practitioners to develop a climate-based dengue early warning systems in Bangladesh. Furthermore, this study recommends community-based surveillance for developing an effective dengue prevention strategy in the dengue endemic cities in Bangladesh.
